# Bibliometric and visualization analysis of mesenchymal stem cells and rheumatoid arthritis (from 2012 to 2021)

**DOI:** 10.3389/fimmu.2022.1001598

**Published:** 2022-10-13

**Authors:** Jiaxi Liu, Jinfang Gao, Qing Niu, Fengping Wu, Zewen Wu, Liyun Zhang

**Affiliations:** ^1^ Third Hospital of Shanxi Medical University, Shanxi Bethune Hospital, Shanxi Academy of Medical Sciences, Tongji Shanxi Hospital, Taiyuan, China; ^2^ School of Basic Medical Sciences, Shanxi Medical University, Taiyuan, China

**Keywords:** visualization analysis, mesenchymal stem cells, rheumatoid arthritis, bibliometric analysis, autoimmune disease

## Abstract

**Background:**

Rheumatoid arthritis (RA) is a chronic autoimmune disease that can lead to joint deformity and loss of function. Recent studies have shown great progress in the research of mesenchymal stem cells (MSCs) in RA. However, thus far, there have been no bibliometric or visualization analyses in this field. This bibliometric analysis provides a comprehensive overview of the general information and research hotspots of MSCs and RA.

**Methods:**

Articles relevant to MSCs and RA, published between 2012 and 2021, were searched using the Web of Science Core Collection database. Irrelevant publications were excluded from the analysis. Bibliometric and visualization analyses were conducted using VOSviewer, CiteSpace, and Scimago Graphica.

**Results:**

A total of 577 articles were analyzed. The annual number of publications increased from 2012 to 2017 and plateaued from 2017 to 2021. China and the USA had the largest number of publications. Collaboration among different organizations mainly occurs between institutes of the same country. Stem Cell Research and Therapy and Frontiers in Immunology were the most popular journals in this field. All the top 20 co-cited authors had a positive co-citation relationship. The top references indicate that MSCs can contribute to RA research and treatment mainly *via* immunomodulation. From 2012 to 2021, “collagen-induced arthritis,” “immunomodulation,” and “therapy” were some of the keywords associated with MSCs and RA, while “extracellular vesicles” showed a strong keyword burst from 2019 to 2021.

**Conclusion:**

MSCs and RA have been widely studied in different countries and institutions and by different authors over the last ten years. China and the USA had the largest number of publications. Different types of journals provide admirable sources for researchers. Some keywords, including immunomodulation and extracellular vesicles, may be hot spots in the near future. There will be more basic research and clinical translation of MSCs and RA, and substantial new treatments for RA will soon be developed.

## Introduction

Rheumatoid arthritis (RA) is a chronic autoimmune disease that can lead to joint deformity and loss of function. Treatments for RA include nonsteroidal anti-inflammatory drugs, corticosteroids, disease-modifying antirheumatic drugs (DMARDs), and biological agents (1). However, not all patients can benefit from these medications. Therefore, there is an urgent need for new methods to treat RA.

Mesenchymal stem cells (MSCs), a promising tool in the research and treatment of RA, can be isolated from a variety of tissues and differentiate into many types of mesodermal lineage cells, such as adipocytes, osteocytes, and chondrocytes (2, 3). MSCs have immense potential for cell therapy because of their immunomodulatory and pluripotency properties (4). In recent years, there have been an increasing number of studies on MSCs in the pathophysiology and treatment of RA (5–7).

Bibliometric analysis is used to analyze the impact of scientific production quantitatively, qualitatively, and visually. This includes obtaining, processing, and managing data from existing publications (8). Details regarding authors, countries, journals, institutions, references, and keywords can be obtained from relevant fields (9).

Several new results on MSCs and RA have been published from 2012 to 2021. Bibliometric and visualization analyses can provide greater insight into publications across years, countries, organizations, journals, and leading teams. Knowledge of the most important references and keywords is important for researchers who wish to understand the history and current status of MSCs and RA. This article offers a panorama of MSCs and RA in the last ten years, which can help researchers, especially beginners, understand the field quickly and effectively to determine promising future directions.

## Methods

### Data source and search strategies

**Figure 1:**
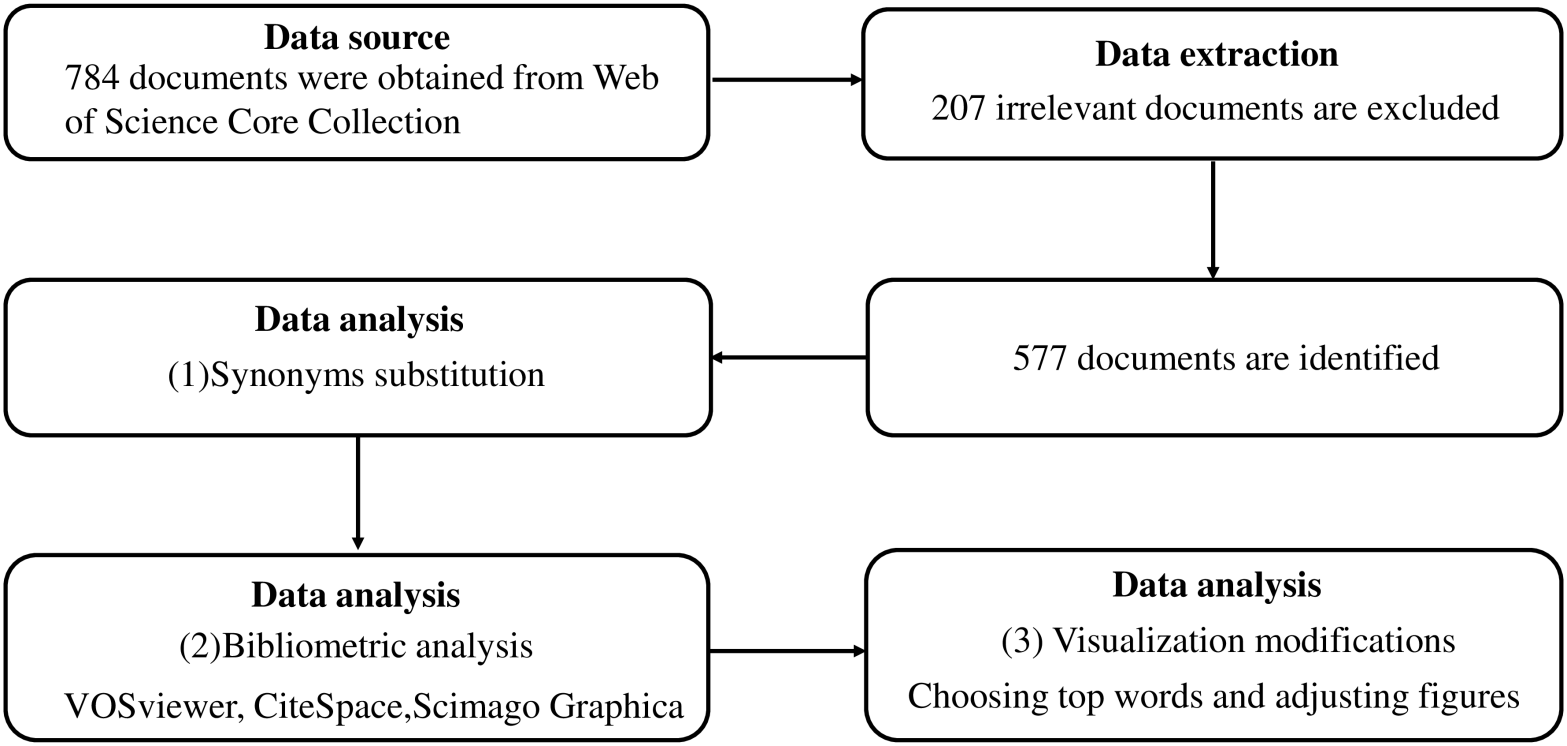
Methods flowchart.

In this article, bibliometric and visualization analyses were conducted through the Science Citation Index Expanded (a sub-field database of the Web of Science Core Collection) on 24 June 2022. The search strategy was as follows: TS = ((“mesenchymal stem cells” OR “MSC” OR “MSCs”) AND (“rheumatoid arthritis” OR “RA”)), and the publication years were from 2012 to 2021. The types of articles were restricted to “articles” and “reviews,” with the language restricted to English. Following these criteria, 17 meeting abstracts, six book chapters, and four non-English papers were excluded in advance, leaving 784 documents that met the criteria (**Figure 1**).

### Inclusion criteria and data extraction

The 784 identified studies were ranked according to relevance on the Web of Science website. Some were deemed irrelevant based on the topic, particularly the documents obtained from the last several webpages. These documents were evaluated by searching the titles, abstracts, and main texts. Studies were included if relevant keywords (such as “mesenchymal stem cells,” “MSCs,” “rheumatoid arthritis,” and “RA”) appeared multiple times and if the main theme was related to MSCs and RA. Similarly, documents were excluded if the relevant keywords appeared only once or twice or if the theme was not relevant to RA or MSCs. For example, some studies have mainly focused on research in osteoarthritis or other fields. In some documents, the keywords in this article (“rheumatoid arthritis” and “mesenchymal stem cells”) were mentioned but were not the focus of the research. Therefore, not all 784 documents were used in this study. After careful review, we selected 577 documents that were most relevant to the topic (**Figure 1**). Subjectivity may exist in the process of inclusion and exclusion, which could lead to selection and publication biases.

### Data analysis

#### Analysis software

VOSviewer (version 1.6.18) is a software that can analyze key information from publications, including countries, organizations, co-citation, and collaboration networks (10), and was used to generate figures showing publications, countries, organizations, top journals, co-cited authors, and keywords.

CiteSpace (Version 6.1.2) is also widely used in bibliometric and visualization analyses (11). In this study, CiteSpace generated figures for top reference citation bursts and keyword bursts.

Scimago Graphica (Version 1.0.23) is a visualization tool for exploring and communicating data. In this study, Scimago Graphica generated a geographical distribution of publications on MSCs and RA.

Microsoft Office Excel 2019 is used to organize data in the analysis process.

#### Synonyms substitution

There were some synonyms among the keywords that may have led to a bias in the results. Therefore, we substituted synonyms, especially words that were closely related to the topic. For example, we merged “mesenchymal stem cells” and “mesenchymal stem cells” before data analysis (**Figure 1**).

#### Visualization modifications

Dozens or even hundreds of results can be generated during analysis. Showing all of these in figures and tables can cause confusion. To help readers effectively and precisely observe trends in the results, we mainly show the top five, top 10, and top 20 results in the figures and tables. In the VOSviewer, the same results can appear in different layouts of dots and clusters. Therefore, the “attraction” and “repulsion” of dots and clusters, which can determine the layouts, were adjusted to obtain better visual effects without changing the results (**Figure 1**).

## Results

### Publications analysis

**Figure 2:**
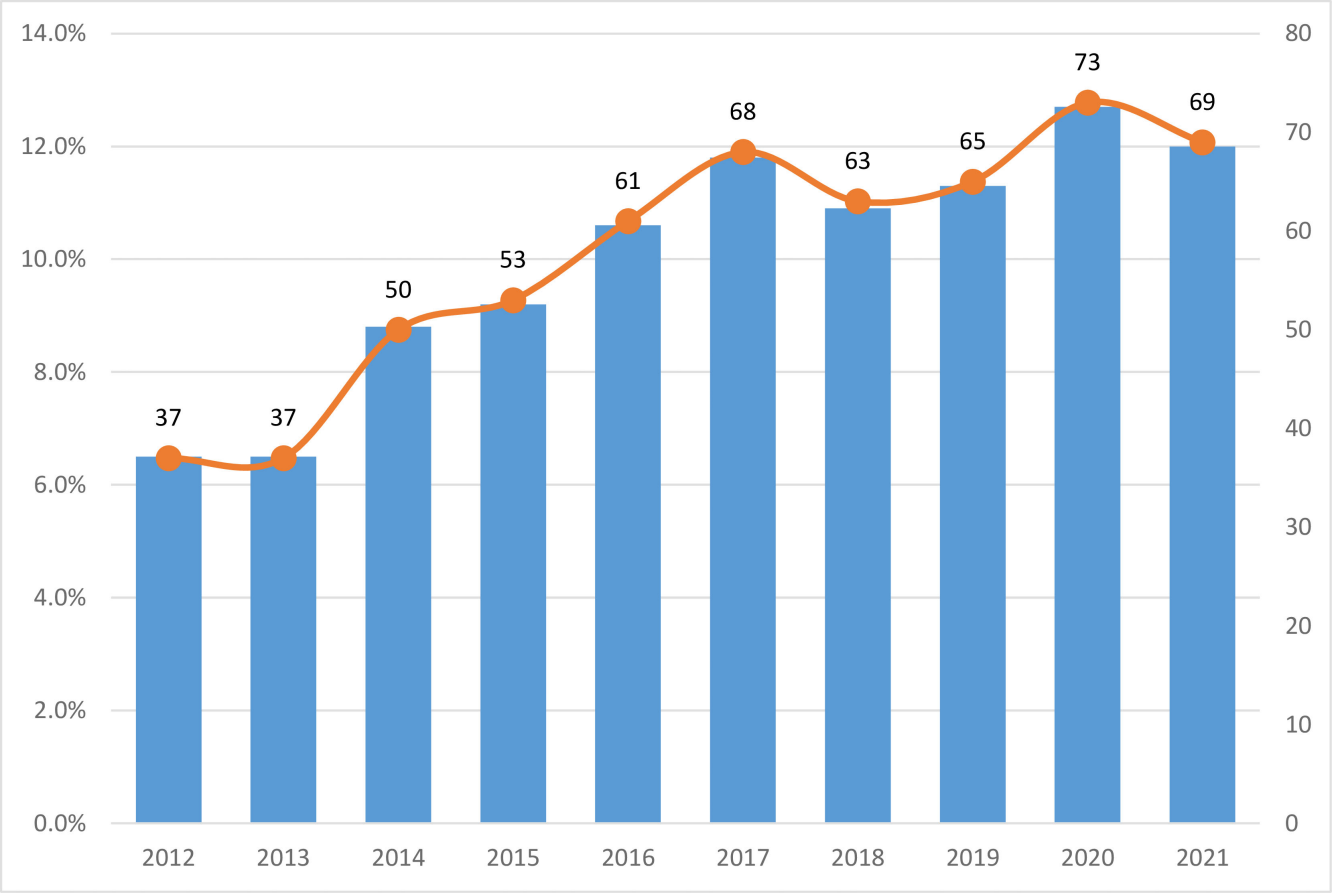
Number of the publications from 2012 to 2021.

As shown in **Figure 2**, there was a steady and rapid increase in the annual number of publications from 2012 to 2017, peaking at 68 publications in 2017. The annual number of publications slightly decreased in 2018 and 2019. In 2020, the number of publications was 73, which hit a record-high. The change from 2017 to 2021 was smaller than that from 2012 to 2017. Due to the large number of publications during the platform period, it may take a few years for a new burst to appear.

### Countries and organizations analysis

**Figure 3:**
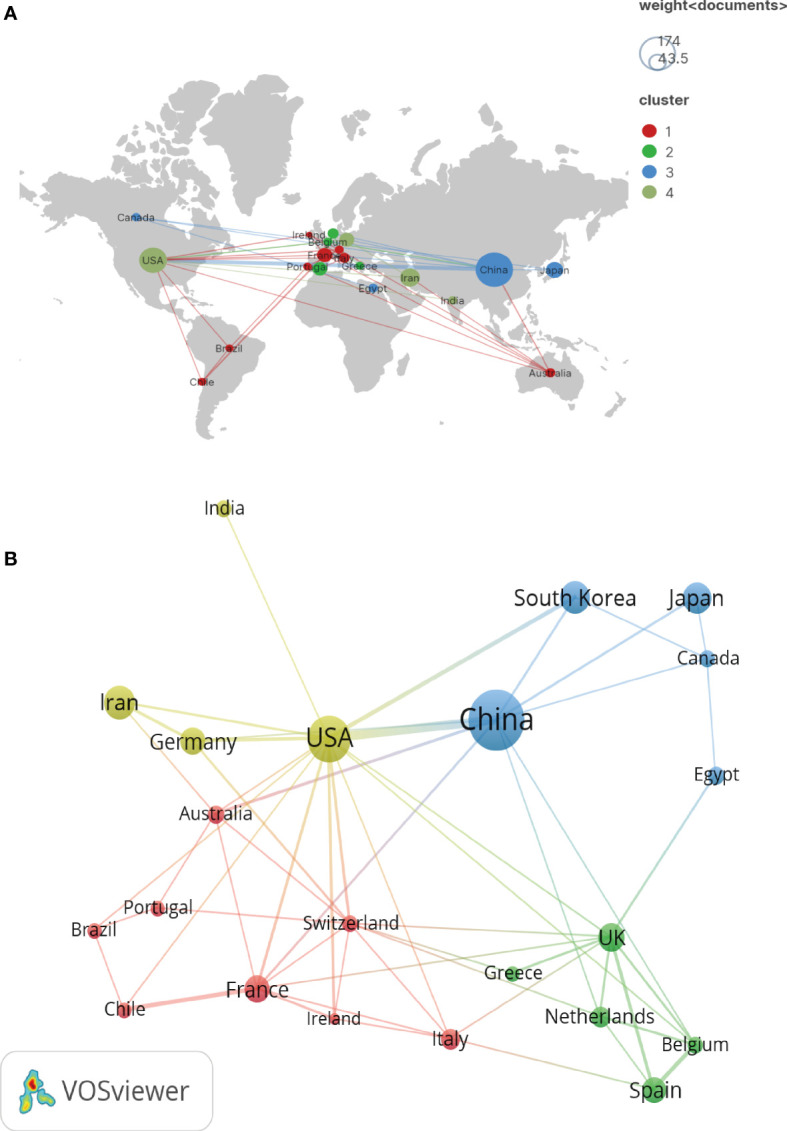
**(A) **The geographical distribution of countries on publications related to MSCs and RA. **(B) **The visualization of countries on publications related to MSCs and RA.

The analyzed publications were from 51 countries and 887 organizations. Countries that published more than five documents were included in the visualization analysis. The specific geographical distribution and partnerships are shown in **Figures 3A, B**. Moreover, some collaborations have been observed between countries. China and the USA published nearly half of the 577 documents and showed the most active collaborations (**Figures 3A, B**, and **Table 1**). Regarding average citations/publications, the USA, Iran, South Korea, and Japan had similar figures (around 25), whereas the average citations per publication of China was slightly lower (18.3) (**Table 1**).

**Table 1:** The publications and citations of Top 5 countries.

Rank	Country	Publications	Citations	Average Citation/Publications
1	China	174	3,187	18.3
2	USA	96	2,834	29.5
3	Iran	48	1,105	23.0
4	South Korea	41	1,064	26.0
5	Japan	37	942	25.6

**Figure 4:**
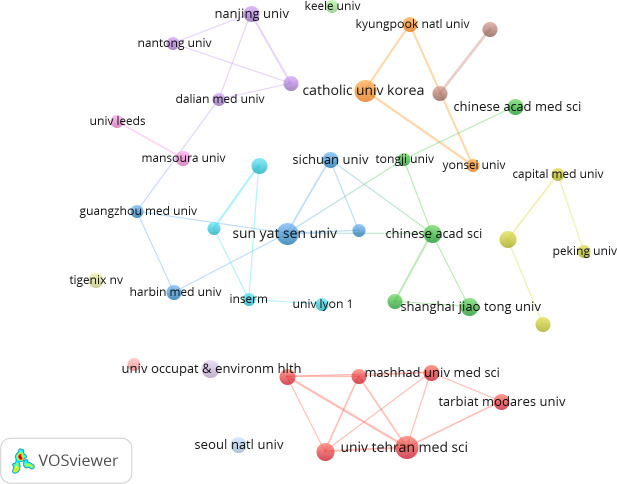
Visualization of organizations related to MSCs and RA publications.

As shown in **Figure 4**, collaboration mainly occurs between different organizations in the same country. The red cluster represents organizations in Iran. The orange cluster represents organizations in South Korea. The green and purple clusters represent organizations in China. The top five organizations are all located in Iran, South Korea, and China (**Table 2**).

**Table 2:** Top 5 organizations in publications and their citations.

Rank	Organization	Publications	Citations
1	Tehran University of Medical Sciences(Iran)	14	350
2	Sun Yat-sen University(China)	13	344
3	Catholic University of Korea(South Korea)	13	242
4	Shanghai Jiao Tong University(China)	9	293
5	Iran University of Medical Sciences(Iran)	9	239

### Top journals

Publications related to MSCs and RA were published in 271 journals. It is worth noting that the Annals of Rheumatic Disease, Nature Reviews Rheumatology, and Autoimmunity Reviews have high citations and publications (more than 50). Compared to the top five journals (**Table 3**) with the largest number of publications, these journals had relatively few publications; nevertheless, they are important resources for research on MSCs and RA.

**Table 3:** Top 5 journals on research of MSCs and RA.

Rank	Journal	IF	Publications	Citations
1	Stem Cell Research & Therapy	8.1	23	593
2	Frontiers in Immunology	8.8	17	671
3	International Journal of Molecular Sciences	6.2	17	405
4	Plos One	3.8	14	440
5	Scientific Reports	5.0	14	360

**Figure 5:**
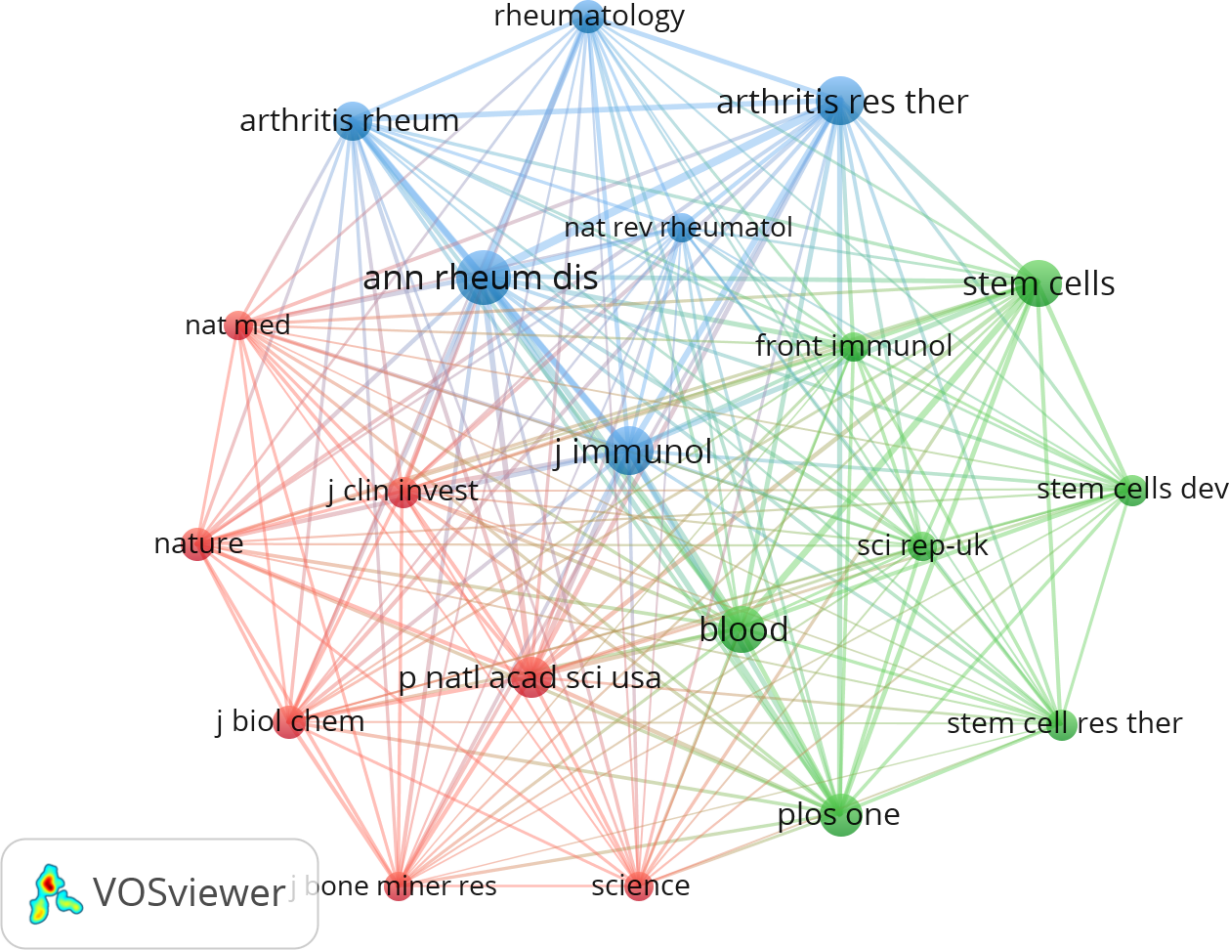
The visualization of co-cited journals on research of MSCs and RA.

Journals with more than 300 co-citations were chosen to generate the top 20 co-cited journal visualization networks (**Figure 5**). All these journals have positive co-citation relationships. The blue cluster represents journals related to rheumatology and arthritis. The green cluster represents journals on stem cells. The red cluster represents the comprehensive journals.

### Co-cited authors analysis

**Figure 6:**
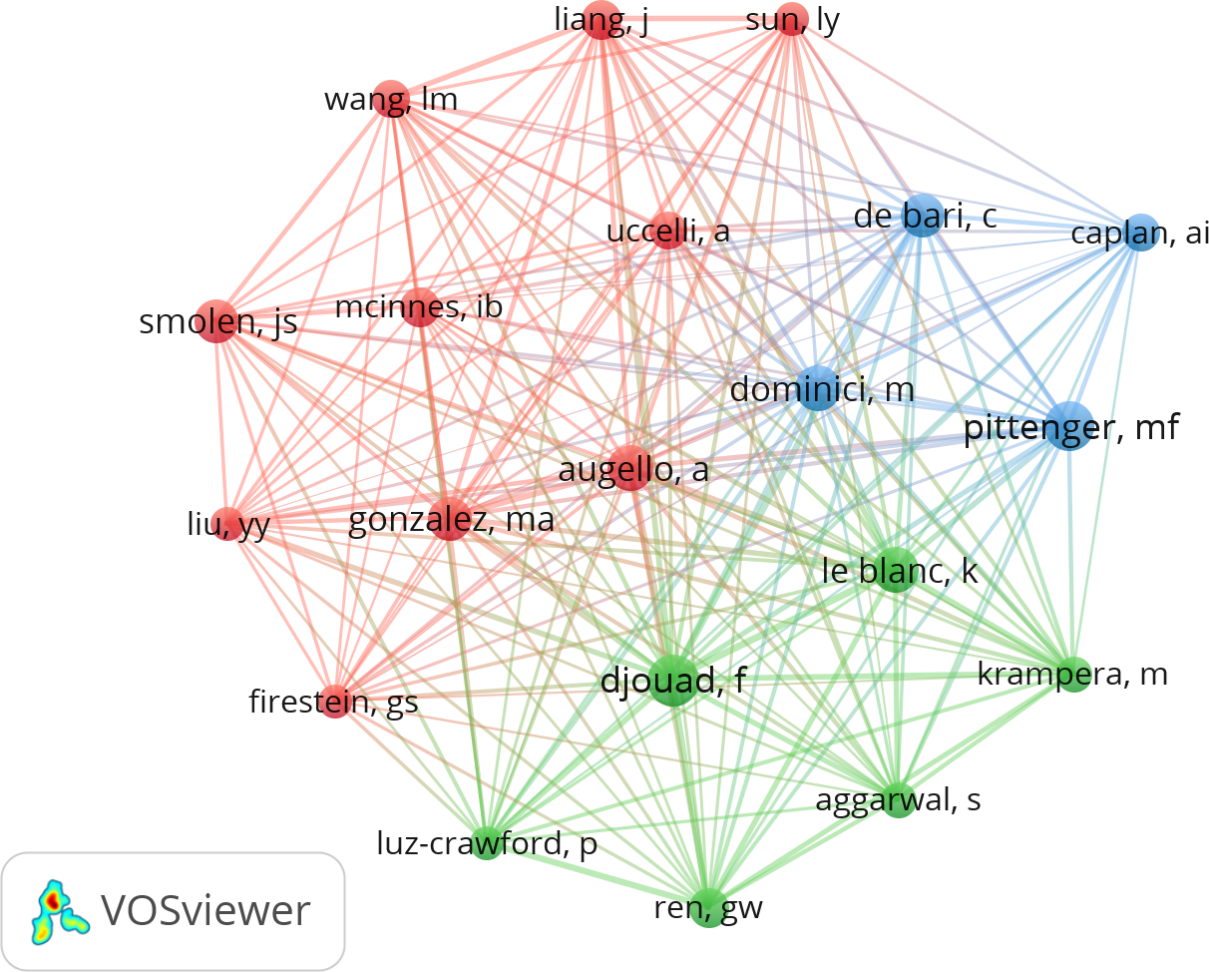
Visualization of Top 20 co-cited authors on research of MSCs and RA.

A co-cited author is defined as a researcher cited by more than two other authors (12). Analysis of the network of the co-authors can reveal which authors contributed the most to the research on MSCs and RA. Among the 20,724 co-authors who contributed to the research on MSCs and RA, 20 authors were co-cited more than 50 times (**Figure 6**). All authors had a positive co-citation relationship with the other authors. The top five co-cited authors with the largest nodes in **Figure 6** were Farida Djouad (from France), Mark F. Pittenger (from the USA), Katarina Le Blanc (from Sweden), Massimo Dominici (from Italy), and Andrea Augello (from the UK).

### Top references

**Figure 7:**
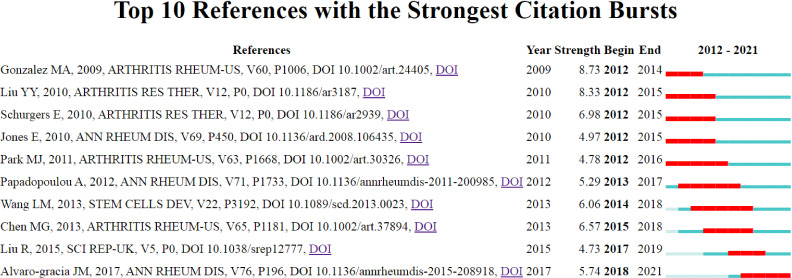
Top 10 references with strong citation bursts. The red bar indicates high citations in that year.

**Figure 7** and **Table 4** show specific information on the top ten references with strong citation bursts. Citation bursts refer to references that are frequently cited by researchers over a period. The red bar represents strong citation bursts. According to the top ten studies, MSCs may benefit RA research and treatment through immunomodulation and regeneration. MSCs can affect both innate and adaptive immune cells, such as macrophages, neutrophils, T cells, and B cells. MSCs can also differentiate into chondrocytes and osteocytes, which ameliorates cartilage and bone destruction in RA. Additionally, MSCs can be obtained from different sources, such as the bone marrow, human umbilical cord, adipose tissue, and synovium.

**Table 4:** The main content of the 10 references with strong citations bursts. The references are ranked by beginning year of burst.

Rank	Main Content	Strength
1	Treatment of experimental arthritis by inducing immune tolerance with human adipose-derived mesenchymal stem cells	8.73
2	Therapeutic potential of human umbilical cord mesenchymal stem cells in the treatment of rheumatoid arthritis	8.33
3	Discrepancy between the *in vitro* and *in vivo* effects of murine mesenchymal stem cells on T-cell proliferation and collagen-induced arthritis	6.98
4	Mesenchymal stem cells in rheumatoid synovium: enumeration and functional assessment in relation to synovial inflammation level	4.97
5	Transforming Growth Factor β-Transduced Mesenchymal Stem Cells Ameliorate Experimental Autoimmune Arthritis Through Reciprocal Regulation of Treg/Th17 Cells and Osteoclastogenesis	4.78
6	Mesenchymal stem cells are conditionally therapeutic in preclinical models of rheumatoid arthritis	5.29
7	Human Umbilical Cord Mesenchymal Stem Cell Therapy for Patients with Active Rheumatoid Arthritis: Safety and Efficacy	6.06
8	Adoptive transfer of human gingiva-derived mesenchymal stem cells ameliorates collagen-induced arthritis *via* suppression of Th1 and Th17 cells and enhancement of regulatory T cell differentiation	6.57
9	Allogeneic mesenchymal stem cells inhibited T follicular helper cell generation in rheumatoid arthritis	4.73
10	Intravenous administration of expanded allogeneic adipose-derived mesenchymal stem cells in refractory rheumatoid arthritis (Cx611): results of a multicenter, dose escalation, randomized, single-blind, placebo-controlled phase Ib/IIa clinical trial	5.74

### Keywords analysis

**Figure 8:**
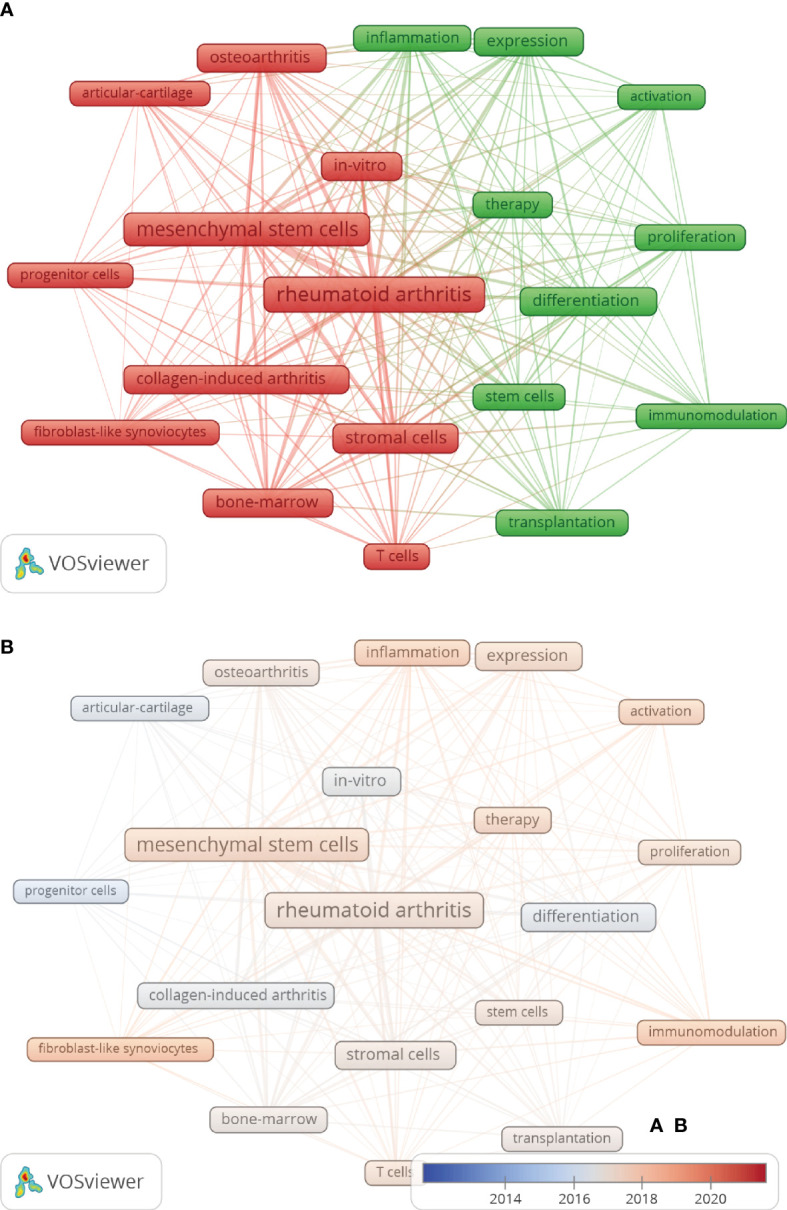
**(A)** Top 20 keywords cluster analysis and trend topic analysis. **(B)** Distribution of average time for top 20 keywords.

A co-occurrence analysis of the keywords was conducted to quickly capture the hotspots in MSCs and RA. **Figure 8A** shows the top 20 high-frequency keywords in the research on MSCs and RA, whereas **Figure 8B** depicts the average time that these keywords appear in.

Among the keywords, “collagen-induced arthritis” represents a typical mouse model of RA research, while “bone-marrow” is one of the main sources of MSCs. The regulation of “T cells” through MSCs has been found to play a vital role in RA pathophysiology and therapy research. The “immunomodulation” of MSCs is the most vital characteristic for RA research and treatment. The “differentiation” and “proliferation” of MSCs are significant for the regeneration of bone and cartilage in RA. Finally, “therapy” indicates that MSCs have been widely studied for RA treatment.

**Figure 9:**
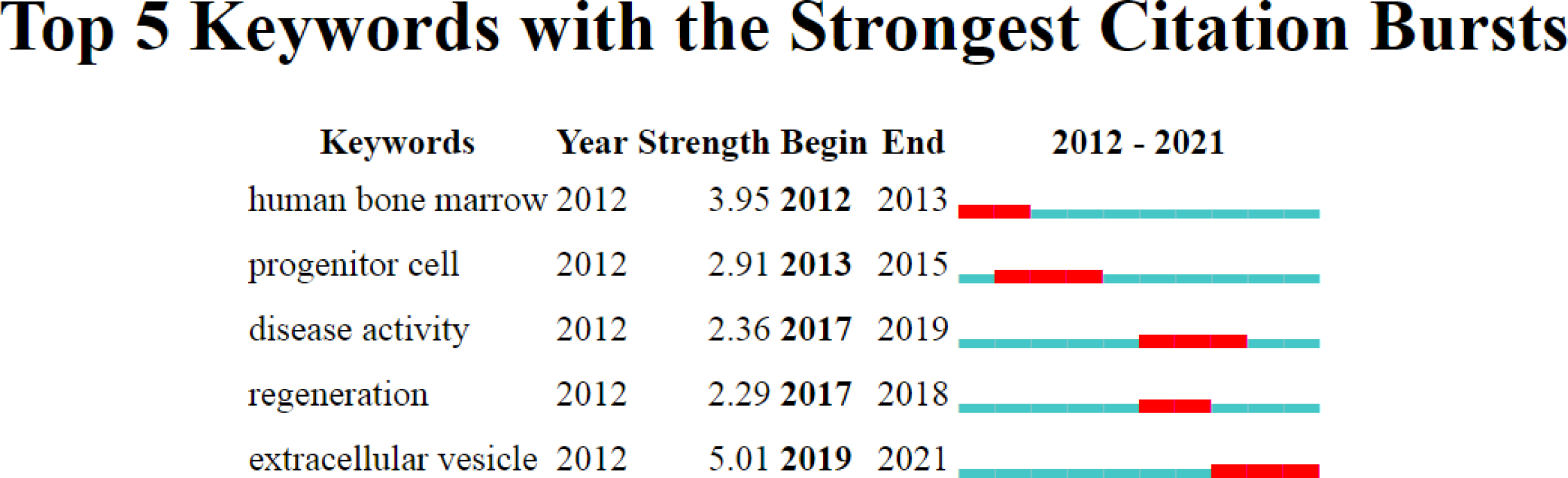
Top 5 keywords with the strong citation bursts. The red bar indicates high citations in that year.

Additionally, it is notable that “extracellular vesicles” showed a strong burst from 2019 to 2021. MSC-derived extracellular vesicles (EVs), including exosomes (13–15), have been hotspots in RA research in recent years (**Figure 9**). MSC-derived exosomes, which are critical paracrine effectors, are small vesicles 30–200 nm in diameter. They play a vital role in cell communication by carrying substances from parental cells, and exert inhibitory effects on various immune cells (16, 17).

## Discussion

A large amount of high-quality research has been conducted over the past decade (2012–2021). In the past five years, approximately 70 articles have been published every year, which is much higher than the number of articles published from 2012 to 2016. Although there are no wide variations in the number of publications, a new burst may appear in “MSCs-derived extracellular vesicles,” “immunomodulation,” and “inflammation” in just a few years. For researchers interested in the cutting-edge knowledge of MSCs and RA, frontiers in immunology and stem cell research and therapy are excellent sources. Although some journals with high citations/publications (more than 50), such as Annals of Rheumatic Disease, Nature Reviews Rheumatology, and Autoimmunity Reviews, do not have copious related publications, they are still valuable references. Different types of journals have been extensively cited according to the results of co-cited journals. Comprehensive journals, journals on rheumatology, immunology, and stem cells are all covered. This signifies that MSCs and RA have attracted wide attention in both basic research and clinical applications. Thus, MSCs treatment may be the most promising treatment for RA in the future.

**Figure 10:**
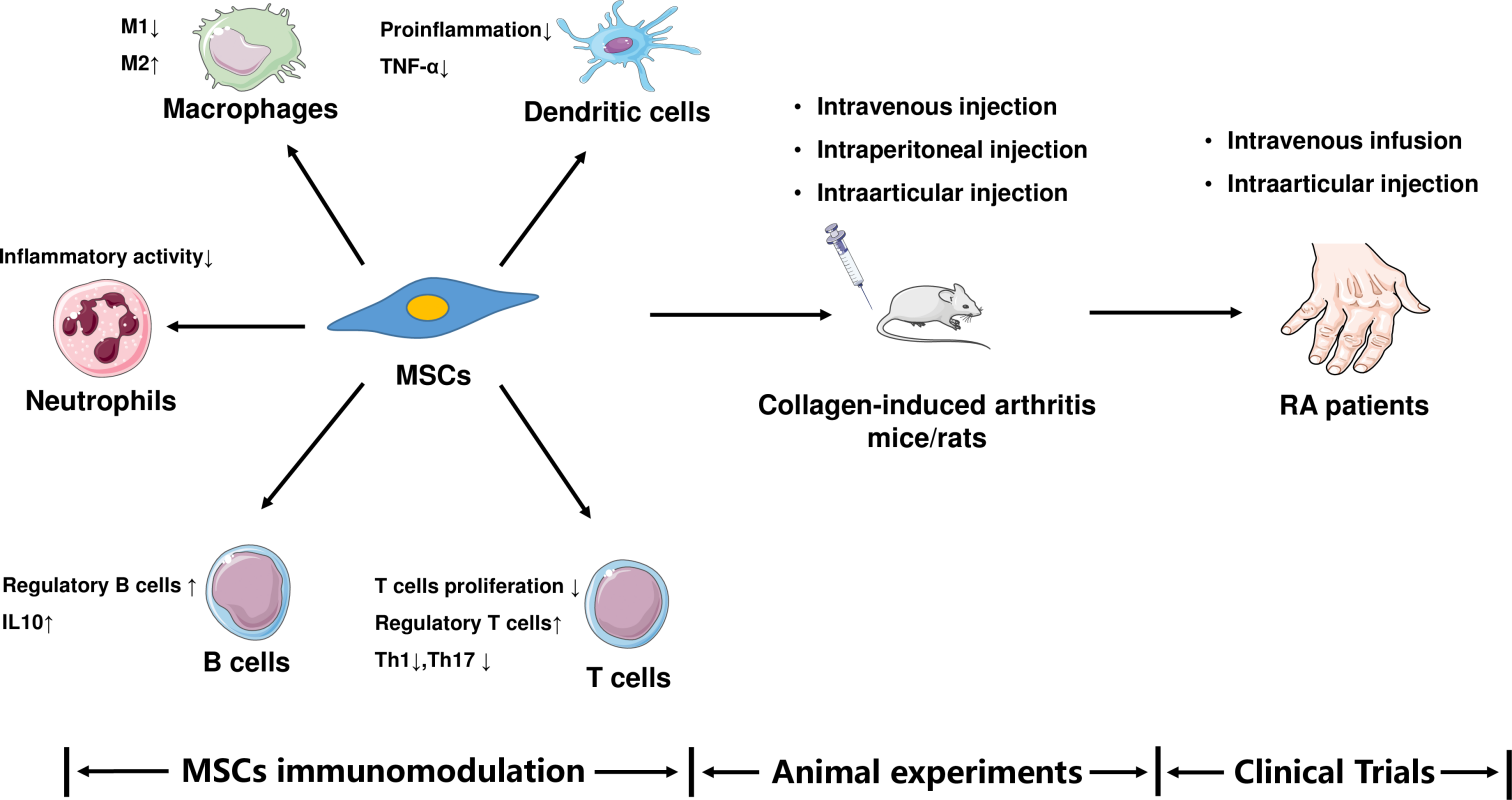
MSCs in RA research.

Immunomodulation of MSCs is the most important characteristic in RA pathogenesis research and treatment (**Figure 10**). MSCs can affect both innate and adaptive immune cells.

Regarding innate immune cells, MSCs promote the differentiation of macrophages from the proinflammatory phenotype M1 to the anti-inflammatory phenotype M2 through secretion of transforming growth factor-β and prostaglandin E2 (18, 19). The phagocytic activity of neutrophils is enhanced by the secretion of IL-17 MSCs. Umbilical tissue-derived MSCs reduce the inflammatory activity of neutrophils (20), while bone marrow MSCs suppress the proinflammatory function of dendritic cells and hamper tumor necrosis factor-α production (21).

Regarding adaptive immune cells, MSCs derived from the human umbilical cord inhibited T-lymphocyte proliferation. In the spleen, MSCs increased the ratio of regulatory T cells and reduced the ratio of Th17 cells. Immunomodulation improves arthritis symptoms and inhibits synovial hyperplasia in collagen-induced arthritis rats (22). Furthermore, adipose tissue-derived MSCs promote the production of regulatory B cells and inhibit plasma cell formation (23), whereas bone marrow MSCs can inhibit Th1 cell and pro-inflammatory cytokine production (21).

An increasing number of studies have been performed using animal models of RA (collagen-induced arthritis rats and mice). In these experiments, MSCs are usually administered *via* intravenous, intraperitoneal, and intra-articular injections (24–29), and the translation from scientific research to clinical practice based on immunomodulation is beginning. According to ClinicalTrials.gov, several researchers have attempted to treat RA by using MSCs. The most relevant clinical trials are currently in phases 1 and 2. Intravenous infusion and intra-articular injections are most commonly used in clinical trials (30–32). MSCs mostly originate from the umbilical cord, bone marrow, and adipose tissues.

RA and osteoarthritis (OA) are the two most common types of arthritis. MSCs are widely used in OA research and treatment (33). There are strong similarities in cell dose and administration route in RA and OA research, although more species and MSC types are used in OA research (2, 7, 34). Studies on RA treatment have shown that MSCs are potentially effective in healing inflammation, reducing bone formation, and reducing disease activity. According to studies on OA treatment, cartilage degeneration was inhibited, inflammatory factors were reduced, and pain was reduced (**Table 5**).

**Table 5:** Comparison of RA and OA in MSCs treatment.

	RA		OA	
	Preclinical	clinical	preclinical	clinical
Species	Mice, rats	Human	Mice, rats, rabbit, dog, sheep	Human
MSC types	Human umbilical cord, murine bone marrow, human adipose tissue, human umbilical cord blood	Allogeneic and autologous adipose tissue, autologous bone marrow, allogeneic umbilical cord	Mice bone marrow, human umbilical cord, human adipose tissue, human amniotic fluid, rats synovial, human synovial fluid	Autologous synovial, autologous bone marrow, allogeneic umbilical cord, allogeneic placental
Cell dose	From 1 ∗ 10^6^ to 5 ∗ 10^6^	From 1 ∗ 10^6^ to 300 ∗ 10^6^	From 0.02 ∗ 10^6^ to 100 ∗ 10^6^	From 1*10^6^ to 200*10^6^
Route of administration	Intraperitoneal, Intravenous, Intra-articular	Intra-articular, Intravenous	Intra-articular Intravenous	Mainly intra-articular
Main findings	Amelioration of inflammation; reduction of bone destruction; decreased disease activity		Inhibited cartilage degeneration; less inflammation factors; reduced pain	

Regarding clinical relevance, MSC treatment can be a great choice for patients with refractory RA and DMARD-resistance. RA occurs more frequently in females than in males (35). Disease activity is usually higher in women, whereas the response rate to DMARDs appears to be higher in men (36). Moreover, sex differences exist in circulating proteins. Women have a higher expression of lipoprotein lipase, interleukin-1 receptor-like 2, and vascular endothelial growth factor D, whereas men showed higher expression of IL-18, Matrix Metallopeptidase 12, and C-reactive protein (37). Therefore, sex-based differences in MSCs treatment are noteworthy, and sex-specific personalized treatments may provide a new direction for MSCs treatment.

According to the keywords in **Figure 8A**, research on MSCs and RA usually starts in “collagen-induced arthritis” mice or rats, which are typical RA animal models. Research in successful animal models has revealed the existence of enormous “fibroblast-like synoviocytes,” which are the most common cell type at the pannus-cartilage junction. “Fibroblast-like synoviocytes” and synovial tissue derived “MSCs” have very similar morphology and surface markers, and the MSCs are probably immature “fibroblast-like synoviocytes” (38). “Fibroblast-like synoviocytes” can also contribute to “articular cartilage” destruction through the production of matrix-degrading molecules, chemokines, and cytokines (39, 40).

The MSCs used for RA research are usually obtained from the “bone marrow.” MSCs play a role in “therapy” *via* “immunomodulation,” “proliferation,” and “differentiation.” A common method for using MSCs is “transplantation.” MSCs are often injected into the articular cavity or infused into the peripheral blood. After MSC treatment, “inflammation” can be suppressed. In addition, the strong link among “rheumatoid arthritis,” “mesenchymal stem cells,” and “osteoarthritis (OA)” indicates that MSCs have been widely used in both RA and OA (**Table 5**).

As shown in **Figure 8B**, the “differentiation” of MSCs and “*in vitro*” cell culture were more popular before 2017, while in research on “immunomodulation” of MSCs, the “fibroblast-like synoviocytes” and “inflammation” have become more popular since 2017. Regarding the rest of the keywords, the distribution of the average time did not change significantly, indicating that they have been consistently studied from 2012 to 2021. In the future, MSC-derived EVs, immunomodulation, fibroblast-like synoviocytes, and inflammation may become trends. Researchers could find new directions and ideas through these possible trends, including the immunomodulation and anti-inflammatory function of MSC-derived extracellular vesicles, as well as transcriptomic and proteomic changes in fibroblast-like synoviocytes at different stages of RA.

Some collaborations can be seen in different countries and continents, which is beneficial for the innovation of research techniques and the extensive application of new findings. Moreover, collaboration among organizations mainly occurs within the same country, where it is more convenient for researchers to communicate with one another. Collaboration among different countries usually involves language, culture, and regulatory barriers, which can complicate research. However, collaborations within the same country may be insufficient. Such barriers can restrict the flow of ideas, delay research progress, and impede the extensive use of new findings. Therefore, collaboration among organizations from different countries is indispensable.

As for the top co-cited authors, Massimo Dominici focuses on MSC-derived small extracellular vesicles and the clinical translation of MSCs. Augello and De Bari conducted extensive studies on MSC-based therapeutic approaches, especially using MSCs derived from synovial membranes. Djouad et al. are involved in the study of tissue and organ regeneration processes. The work by Pittenger is mainly about MSC immunology and treatment. The team of Katarina Le Blanc concentrated on experimental and clinical studies of the immunological properties of fetal and adult MSCs. This team is also the first to report that adoptive transfer of MSCs alleviates treatment-resistant acute graft-versus-host disease. The work by González was about experimental arthritis treatment using adipose-derived MSCs. Each team has an area of expertise. Researchers can check the updates of teams with similar research content. This encourages researchers to enrich their knowledge and gain new ideas.

From the analysis of the co-cited authors, we found that the authors came from different organizations in different countries and continents. This is favorable because diversity can provide different perspectives and add various results to MSCs and RA research. Moreover, it is important that the conclusions drawn by one group can be examined or applied to other groups from other countries or continents. This increases the reliability of conclusions and benefits more people worldwide.

In **Figure 7** and **Table 4**, the top ten references with strong citation bursts are listed by the beginning year of the burst. In the first few years, references are mainly about animal experiments and *in vitro* cell experiments, such as “Therapeutic potential of human umbilical cord mesenchymal stem cells in the treatment of rheumatoid arthritis” and “Mesenchymal stem cells in rheumatoid synovium: enumeration and functional assessment in relation to synovial inflammation level”. In the last few years, more articles related to clinical translation have emerged, such as “Human Umbilical Cord Mesenchymal Stem Cell Therapy for Patients with Active Rheumatoid Arthritis: Safety and Efficacy” and “Intravenous administration of expanded allogeneic adipose-derived mesenchymal stem cells in refractory rheumatoid arthritis (Cx611): results of a multicenter, dose escalation, randomized, single-blind, placebo-controlled phase Ib/IIa clinical trial”. Accordingly, an increasing number of clinical studies will be conducted, and large-scale use of MSCs for RA treatment may soon come.

This study had several advantages. For the first time, we systematically analyzed research on MSCs and RA using bibliometrics to provide guidance to researchers working in related fields. Thus, visualization analysis is more explicit, which allows readers to effectively capture key information. This study has several limitations. First, the data were extracted from the Web of Science Core Collection database, which may have missed some relevant information. Second, we chose data published in English; thus, we could not show trends regarding the non-English data. Furthermore, the data extraction protocol may have led to a bias.

## Conclusion

Over the last 10 years, MSCs have become increasingly important in the treatment and pathophysiology of RA. Many publications have indicated that research on MSCs and RA is highly valued by researchers and institutions worldwide. China and the USA had the largest number of publications. Meanwhile, cooperation exists between different countries. Collaboration among institutions mainly occurs in the same country, and more collaborations from different countries are expected. Different types of journals, especially Frontiers in Immunology and Stem Cell Research and Therapy, provide admirable sources for researchers. The top co-cited authors from different countries and continents have their respective areas of expertise and contribute to the diversity and popularization of MSC and RA research. From 2017 to 2021, more articles about clinical translation have been seen in strong citation burst references than from 2012 to 2016. The top 20 keywords covered animal models (CIA mice or rats), sources of MSCs (bone marrow, adipose, etc.), vital characteristics of MSCs in RA research (immunomodulation, proliferation, and differentiation), and pathophysiological changes in RA (such as articular cartilage destruction and fibroblast-like synoviocyte proliferation). In addition, some keywords, including immunomodulation and extracellular vesicles, may be hot spots in the near future. In the future, there will be more basic research and clinical translation of MSCs and RA, and substantial new treatments for RA will soon be developed.

## Data availability statement

The original contributions presented in the study are included in the article/supplementary material. Further inquiries can be directed to the corresponding author.

## Author contributions

JL and JG wrote the manuscript. JL and JG have contributed equally to this work and share first authorship. ZW, FW, QN, and JL analyzed the data and drew pictures. LZ reviewed and revised the manuscript. All authors approved the final manuscript.

## Funding

This work is supported by the National Natural Science Foundation of China (grant number 81771768).

## Acknowledgments

We thank SERVIER MEDICAL ART and all authors who participated in the study of MSCs and RA.

## Conflict of interest

The authors declare that the research was conducted in the absence of any commercial or financial relationships that could be construed as a potential conflict of interest.

## Publisher’s note

All claims expressed in this article are solely those of the authors and do not necessarily represent those of their affiliated organizations, or those of the publisher, the editors and the reviewers. Any product that may be evaluated in this article, or claim that may be made by its manufacturer, is not guaranteed or endorsed by the publisher.
